# Multidrug-resistant *Neisseria gonorrhoeae* isolate SE690: mosaic *penA-60.001* gene causing ceftriaxone resistance internationally has spread to the more antimicrobial-susceptible genomic lineage, Sweden, September 2022

**DOI:** 10.2807/1560-7917.ES.2023.28.10.2300125

**Published:** 2023-03-09

**Authors:** Daniel Golparian, Nora Vestberg, Wiktor Södersten, Susanne Jacobsson, Makoto Ohnishi, Hong Fang, Karin Haij Bhattarai, Magnus Unemo

**Affiliations:** 1 WHO Collaborating Centre for Gonorrhoea and Other STIs, National Reference Laboratory for STIs, Department of Laboratory Medicine, Faculty of Medicine and Health, Ӧrebro University, Ӧrebro, Sweden; 2 Department of Clinical Microbiology, Karolinska University Hospital, Huddinge, Sweden; 3 Department of Venerology at Karolinska University Hospital, Stockholm, Sweden; 4 Department of Bacteriology I, National Institute of Infectious Diseases, Tokyo, Japan; 5 Institute for Global Health, University College London (UCL), London, United Kingdom

**Keywords:** *Neisseria gonorrhoeae*, ceftriaxone, multidrug-resistant *N. gonorrhoeae* (MDR-NG), antimicrobial resistance, *penA-60*, Sweden, travel

## Abstract

We report a ceftriaxone-resistant, multidrug-resistant urogenital *Neisseria gonorrhoeae* in a female sex worker in Sweden, September 2022, who was treated with ceftriaxone 1 g, but did not return for test-of-cure. Whole genome sequencing of isolate SE690 identified MLST ST8130, NG-STAR CC1885 (new NG-STAR ST4859) and mosaic *penA-60.001*. The latter, causing ceftriaxone resistance in the internationally spreading FC428 clone, has now also spread to the more antimicrobial-susceptible genomic lineage B, showing that strains across the gonococcal phylogeny can develop ceftriaxone resistance.

The emerging resistance in *Neisseria gonorrhoeae* (NG) to the last remaning option for empiric monotherapy of gonorrhoea, ceftriaxone, is a global public health concern. We report a ceftriaxone-resistant, multidrug-resistant NG strain in a female sex worker (FSW) in Sweden, September 2022. 

## Clinical case description and diagnosis

In September 2022, an asymptomatic FSW in her 20s (from an eastern European country) attended a sexually transmitted infections (STI) healthcare centre in Sweden for STI screening because she had had multiple unprotected sexual contacts. Vaginal and oropharyngeal swabs were positive for NG in nucleic acid amplification tests (NAATs; BD ProbeTec ET CTQ^x^/GCQ (Becton, Dickinson and Company (BD), Franklin Lakes, United States (US)) on BD Viper with confirmation using BD CTGCTV2 on BD MAX). Testing for *Chlamydia trachomatis* was negative. Approximately 2.5 weeks later, the woman returned for treatment with ceftriaxone 1 g. Urethral swab was then NG culture-positive (isolate SE690), while cervical and oropharyngeal swabs were NG culture-negative. No sexual contacts could be traced and the woman did not return for test-of-cure because she left Sweden.

## Culture and antimicrobial susceptibility testing

Isolate SE690 was cultured from a urethral swab on modified Thayer–Martin agar at +36 ± 1 °C in a humid 5% CO_2_-enriched atmosphere. Species verification was performed by matrix-assisted laser desorption/ionization-time of flight (MALDI-TOF) mass spectrometry (Vitek MS, bioMérieux, Marcy-l'Étoile, France). Minimum inhibitory concentrations (MIC) were determined by Etest (bioMérieux) for 10 antimicrobials and by agar dilution for zoliflodacin and lefamulin (Table). The isolate was resistant to ceftriaxone, cefixime, cefotaxime, ciprofloxacin, tetracycline and benzylpenicillin but susceptible to azithromycin and spectinomycin. The MIC of gentamicin, ertapenem and the new antimicrobials zoliflodacin [1,2] and lefamulin [3-5] were considered wild-type according to previous publications and because no known antimicrobial resistance (AMR) determinants for these antimicrobials were detected (Table).

**Table:** Minimum inhibitory concentrations of antimicrobials for the ceftriaxone-resistant, multidrug-resistant *Neisseria gonorrhoeae* isolate SE690, Sweden, September 2022

Antimicrobial	MIC (mg/L)	Interpretation (EUCAST v 13.0 [25])
Ceftriaxone	0.25	Resistant
Cefixime	1	Resistant
Cefotaxime	1	Resistant
Ciprofloxacin	8	Resistant
Tetracycline	32	Resistant
Benzylpenicillin	> 32	Resistant
Azithromycin	0.5	Susceptible^a^
Spectinomycin	16	Susceptible
Gentamicin	8	NA (wild-type MIC)
Ertapenem	0.016	NA (wild-type MIC)
Zoliflodacin^b^	0.064	NA (wild-type MIC)
Lefamulin^c^	0.125	NA (wild-type MIC)

MIC: minimum inhibitory concentration; EUCAST: European Committee on Antimicrobial Susceptibility Testing; NA: not applicable (due to lack of breakpoints for interpretation).

^a^ Because clinical EUCAST breakpoints are lacking for azithromycin, EUCAST’s epidemiological cut-off (ECOFF) value of MIC > 1 mg/L for azithromycin was used to distinguish susceptibility from resistance [25].

^b^ Pre-licensing international phase III randomised clinical trial [1,2] will finish in 2023.

^c^ Novel antimicrobial with high in vitro activity against *N. gonorrhoeae* [3-5].

## Molecular investigation

Bacterial DNA was isolated using QIAsymphony (QIAGEN, Hilden, Germany) with the DSP DNA Midi Kit (QIAGEN). Next-generation sequencing (NGS) using Illumina (San Diego, US) and Oxford Nanopore Technologies (Oxford, United Kingdom (UK)), and bioinformatic analysis were performed as previously described [4,6,7].

Isolate SE690 was assigned to multilocus sequence typing (MLST) sequence type (ST) 8130, the novel NG sequence typing for AMR (NG-STAR) ST4859 and NG-STAR clonal complex (CC) 1885. The resistance to ceftriaxone, cefixime and cefotaxime was associated with the mosaic *penA-60.001* allele, which also causes ceftriaxone resistance in the internally spreading NG FC428 clone [8-14], AT159 [6,7] and WHO Q [15]. In addition, the SE690 finished chromosome contained additional chromosomal AMR determinants, e.g. GyrA S91F/D95A, ParC S87N/E91Q and *rpsJ* V57M, and plasmids carrying *tetM* and *bla*_TEM-135_, which caused the high-level resistance to ciprofloxacin, tetracycline and benzylpenicillin (Table) [16]. Isolate SE690 additionally harboured the NG cryptic plasmid.

**Figure 1:**
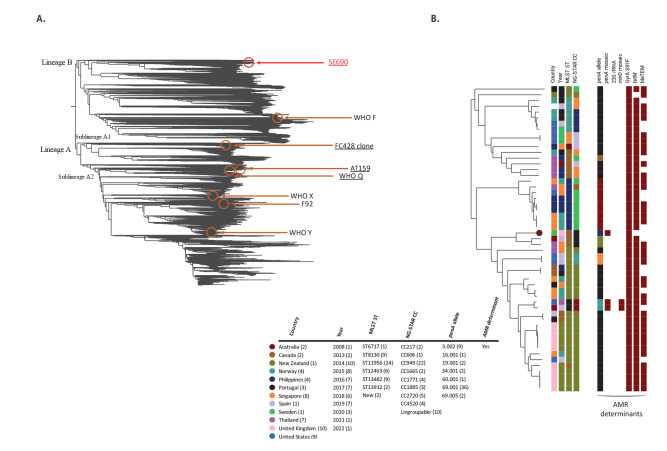
Core phylogenomics of publicly available *Neisseria gonorrhoeae* genome sequences (n = 38,370) showing the ceftriaxone-resistant, multidrug-resistant SE690 isolate from Sweden, September 2022

AMR: antimicrobial resistance; CC: clonal complex; MLST: multilocus sequence typing; ST: sequence type; NG-STAR: *Neisseria gonorrhoeae* sequence typing for antimicrobial resistance; WHO: World Health Organization.

Phylogenomic analysis including publicly available NG genomes (n = 38,370) showed that SE690 differs greatly from previously reported ceftriaxone-resistant, multidrug-resistant (MDR) NG strains (Figure 1A). For example, mosaic *penA-60.001*-containing ones such as the internationally spreading FC428 clone [8-14], AT159 [6,7] and WHO Q [15], but also WHO X [17], WHO Y [17] and F92 [4], are located in genomic lineage A and very distant to SE690 (> 4,000 single nucleotide polymorphisms (SNP)) in genomic lineage B (Figure 1A). 

Panel A shows the phylogenomic relationship of the SE690 isolate and 38,370 publicly available gonococcal genomes. The tree shows SE690 in the more antimicrobial-susceptible lineage B and previously reported ceftriaxone-resistant strains harbouring mosaic *penA*-60.001 (underlined) or other ceftriaxone-resistance determinants (all in lineage A). WHO F [17] represents strains susceptible to most antimicrobials.

Panel B shows a subtree including SE690 (dark red circle) and the 52 most closely related isolates. Coloured bars represent country of infection when available (otherwise reporting country), year, MLST ST, NG-STAR CC, *penA* allele and presence of other key AMR determinants. Data or AMR determinant lacking when blank bars.

Publicly available gonococcal genomes (n = 38,370) were downloaded from the European Nucleotide Archive using the search term ‘Taxon:485’ (12 December, 2022) and analysed as described [4,6]. All genomes were characterised based on a core genome MLST (cgMLST) scheme defined by the 2016 WHO gonococcal reference strains [17], WHO Q [15] and FC428 [8] using Ridom Seqsphere+ (v.8.4.1). Pairwise comparisons of the cgMLST alleles (n = 2,062) were performed and a subsequent neighbour-joining tree was constructed in Ridom Seqsphere+ (v.8.4.1). Phylogenomic trees and subtrees were visualised in Microreact (https://microreact.org/). Isolates closely related to SE690 (n = 52) were characterised for AMR determinants and typing schemes using CLC Genomics Workbench (v22.0.1) as described [6,26].

**Figure 2:**
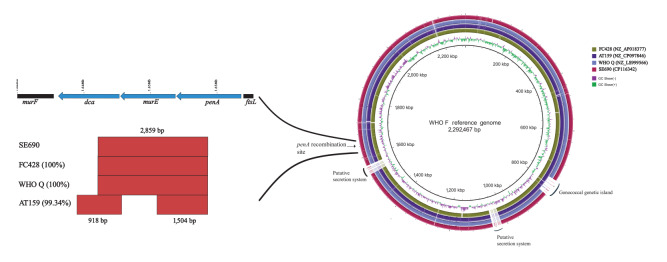
BLAST atlas of the finished *Neisseria gonorrhoeae* genomes of the ceftriaxone-resistant strains WHO Q, AT159, FC428 and the ceftriaxone-resistant, multidrug-resistant SE690 isolate from Sweden, September 2022

A detailed comparison of the finished genomes of SE690 and other ceftriaxone-resistant mosaic *penA-60.001*-containing strains (FC428 strain [8], AT159 [6,7] and WHO Q [15]) showed that SE690 shares an identical 4,875 bp sequence (spanning *dca* (1,647 bp), *murE* (1,479 bp) and *penA* (1,749 bp)) with FC428 [8] and WHO Q [15], which includes one predicted recombination event (2,859 bp sequence). A nearly identical (99.34%) sequence was found also in AT159 [6,7], in which two recombination events were predicted (Figure 2). The identical region in SE690, FC428 [8] and WHO Q [15] was predicted to be due to a recombination event spanning parts of *dca* (540 bp; 32.8%), the entire *murE* gene, and parts of *penA* (840 bp; 48% of the gene) (Figure 2).

WHO: World Health Organization.

BLAST atlas of the finished *N. gonorrhoeae* genomes of the ceftriaxone-resistant strains SE690, WHO Q [15], AT159 [6,7] and FC428 [8] compared with the reference genome of WHO F [17] (not shown for improved visualisation). The arrow shows the *penA* recombination site for all four isolates carrying mosaic *penA-60.001*. The figure on the left hand side is a schematic of the predicted recombination (red blocks) leading to the acquisition of *penA-60.001* in the four isolates using gubbins (https://github.com/nickjcroucher/gubbins) with default parameters, and in paranthesis is the similarity score to SE690 in the region. The WHO F annotation on top shows the three genes involved in the recombination event including *penA*.

A phylogenomic examinination of SE690 and the 52 most closely related genomes (Figure 1B) showed that many related isolates were reported from the UK (n = 10) and the US (n = 9), followed by Singapore (n = 8) and Thailand (n = 7). The most common MLST ST and NG-STAR CC was ST11956 (45.3%) and CC949 (41.5%), respectively. However, the two most closely related isolates belonged to NG-STAR CC1885 (from Australia, 2019 and Thailand, 2018), differing by 198 and 224 SNP, respectively. As most isolates in genomic lineage B, determinants for resistance to currently recommended antimicrobials, such as ceftriaxone and cefixime (mosaic *penA*) and azithromycin (23S rRNA gene mutations and mosaic *mtrRCDE*), were rare in these 53 genomes. However, all isolates had GyrA S91F, and most isolates harboured *bla*_TEM_ (71.7%) and *tetM* (96.2%), which are common in genomic lineage B.

## Discussion

Resistance to ceftriaxone has been exceedingly rare during recent years in Europe (0.03% in 2020) [18] and Sweden (0% in 2015–2021) [19]. However, in 2013 and 2014, sporadic ceftriaxone resistance and verified treatment failures were documented in Sweden [19,20].

In a nation-wide genomic epidemiological study in Sweden, including 1,279 isolates from 2016 [21], only one isolate with the same NG-STAR CC as SE690 (CC1885) was found. As SE690, this isolate belonged to genomic lineage B and the isolate was cultured from a young male who had been infected in Thailand. In the present study, six of the isolates most closely related to SE690 were cultured from 20–52 years-old males infected in Thailand (n = 3) or the Philippines (n = 3). These isolates had a similar antimicrobial susceptibility profile for azithromycin, ciprofloxacin and spectinomycin, but they were highly susceptible to ceftriaxone. Unfortunately, data regarding country of infection is lacking for SE690 and many other closely related isolates, and while many of these isolates have been reported from Europe and North America, an initial importation from Asia cannot be excluded. 

It is of concern that SE690 is the first isolate with mosaic *penA-60.001*, causing ceftriaxone resistance [6-14], in genomic lineage B, a lineage originally characterised by NG isolates susceptible to currently recommended therapeutic antimicrobials. The ceftriaxone-resistant SE690 strain may have emerged through a single horizontal gene transfer (HGT) of a 2,859 bp sequence, including segments of the *penA* gene resulting in mosaic *penA-60.001,* to a ceftriaxone-susceptible MLST ST8130 and/or NG-STAR CC1885 strain. The donor of this 2,859 bp sequence could be a ceftriaxone-resistant NG strain such as FC428 [8], which includes an identical sequence. However, it is more likely that both SE690 and FC428 [8] emerged independently by HGT of the identical sequence and from the same donor such as *N. cinerea* [22] or *N. subflava* [23]. Nevertheless, multiple HGT events cannot be completely excluded.

It is a concern that mosaic *penA-60.001*-containing ceftriaxone-resistant strains are now found in both the genomic lineage A and the more antimicrobial-susceptible genomic lineage B, which illustrates that both ceftriaxone-resistant strains and ceftriaxone resistance-mediating sequences are spreading. The vast majority of these ceftriaxone-resistant isolates have been reported in Asia or in patients infected in Asia [6-15], where ceftriaxone resistance is high in several countries [24]. The woman infected by SE690 left Sweden before test-of-cure and no sexual contacts were traced. The biofitness of SE690 remains unknown, however, FC428 with its identical ceftriaxone resistance-mediating mosaic *penA-60.001* has been shown to have an adequate biofitness [11,23].

## Conclusions

The mosaic *penA-60.001-*containing sequence, causing ceftriaxone resistance in the internationally-spreading FC428 clone, WHO Q and AT159, has now spread to the more antimicrobial-susceptible genomic lineage B. Strains across the NG species phylogeny have shown their ability to develop ceftriaxone resistance through possibly a single HGT, which is a public health concern. It is imperative to increase the awareness of sporadic ceftriaxone-resistant cases, especially imported from Asia, and to enhance the AMR surveillance in Europe and globally, ideally including NGS, particularly in many Asian countries. Most importantly, improved prevention (including condom use), early diagnosis and treatment (including test-of-cure, sexual contact tracing and treatment of index case and contact) and increased focus on and testing of groups at higher risk for STIs, including sex workers and their clients, are imperative. Ultimately, novel, effective and affordable antimicrobials for the treatment of gonorrhoea are essential. 
